# Supplementary material for: Sustained IFN signaling is associated with delayed development of SARS-CoV-2-specific immunity
Source: Nat Commun. 2024 May 16;15:4177. doi: 10.1038/s41467-024-48556-y (PMC11522391; doi:10.1038/s41467-024-48556-y)
Supplement: Supplementary file 1 — Supplementary Information [file 41467_2024_48556_MOESM1_ESM.pdf]

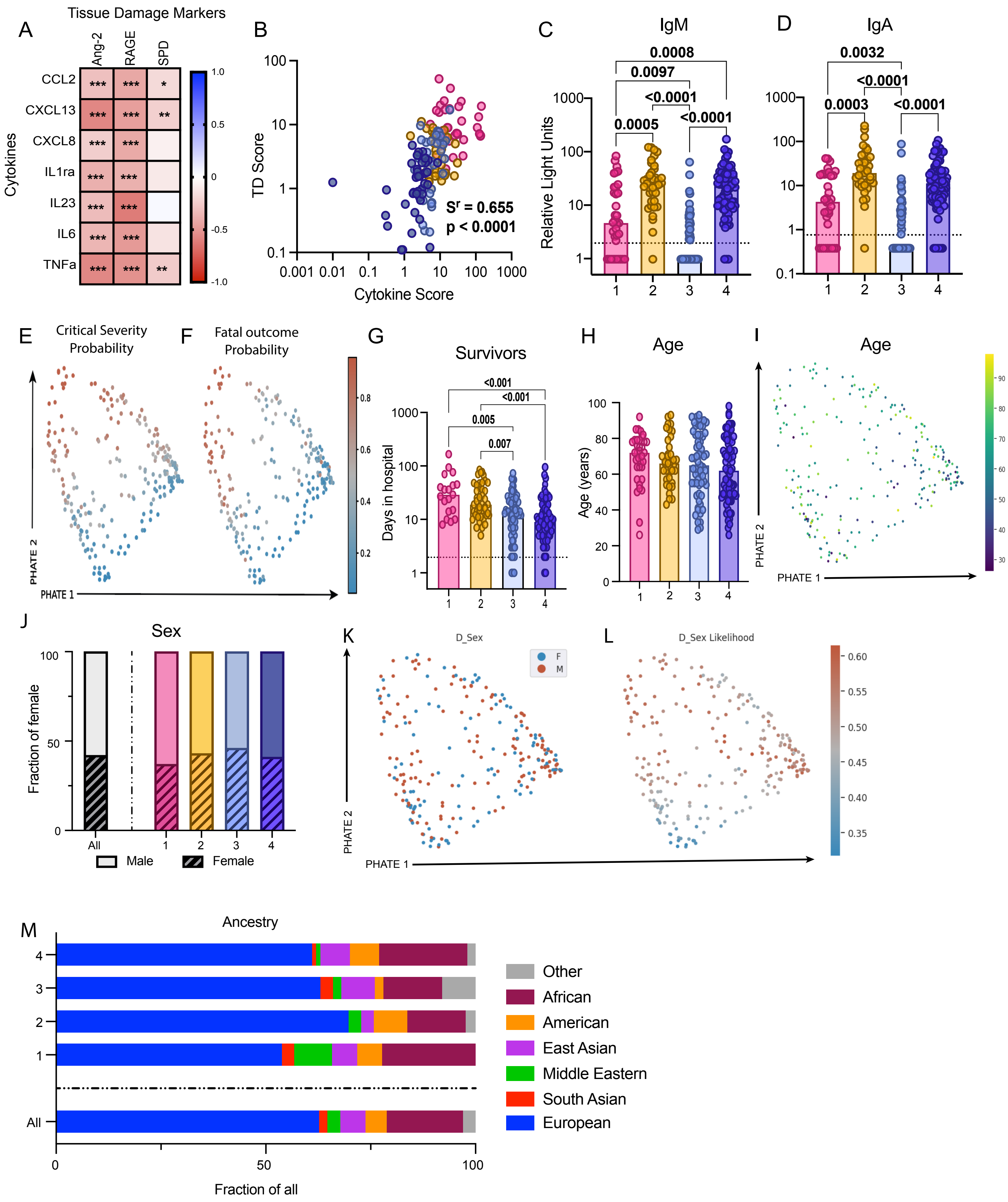
A
Tissue Damage Markers
Ang-2
RAGE
SPD
Cytokines
CCL2
CXCL13
CXCL8
IL1ra
IL23
IL6
TNFa
1.0
0.5
0
-0.5
-1.0
B
TD Score
Cytokine Score
Sʳ = 0.655
p < 0.0001
C
IgM
Relative Light Units
0.0008
0.0097
<0.0001
<0.0001
0.0005
D
IgA
0.0032
<0.0001
0.0003
<0.0001
E
Critical Severity
Probability
F
Fatal outcome
Probability
PHATE 1
PHATE 2
G
Survivors
Days in hospital
<0.001
<0.001
0.005
0.007
H
Age
Age (years)
I
Age
J
Sex
Fraction of female
All
Male
Female
K
D_Sex
F
M
L
D_Sex Likelihood
M
Ancestry
Fraction of all
Other
African
American
East Asian
Middle Eastern
South Asian
European

S1

Supplementary Figure 1. Demographics are not differentially associated with patient clusters. A) Correlation matrix of cytokines and tissue damage markers as measured in all patients of the discovery cohort at DSO 11. **B)** Correlation between cytokine scores calculated on the seven cytokines, with the tissue damage (TD) score, calculated on the three tissue damage markers. **CD**) At DSO11, plasma concentration across four patient clusters of RBD-specific **C**) IgM or **D**) IgA. **EF**) MELD representations of local enrichment across PHATE embedding, of E) critical severity or F) fatal outcome. **G**) Days in hospital among survivors of patient clusters. **HI)** Age of patients H) per patient cluster or I) over the PHATE embedding. **J)** Percentage of the whole cohort or per patient cluster which are female (hashed). **K)** Datapoints are color-coded based on sex of patient across PHATE embedding. **L**) MELD representations of the probability of a region of the embedding begin dominated by males (red) or females (blue). **M**) Fraction of dominant ancestry per patient cluster, calculated from genotyping. N discovery cohort: 1 = 38; 2 = 49; 3 = 73; 4 = 82 (242 in total). AB) Spearman R. * $p< 0.05$; ** $p < 0.01$; *** $p < 0.001$. Kruskal-Wallis with Dunn's multiple comparison tests. JM) Chi2 tests.

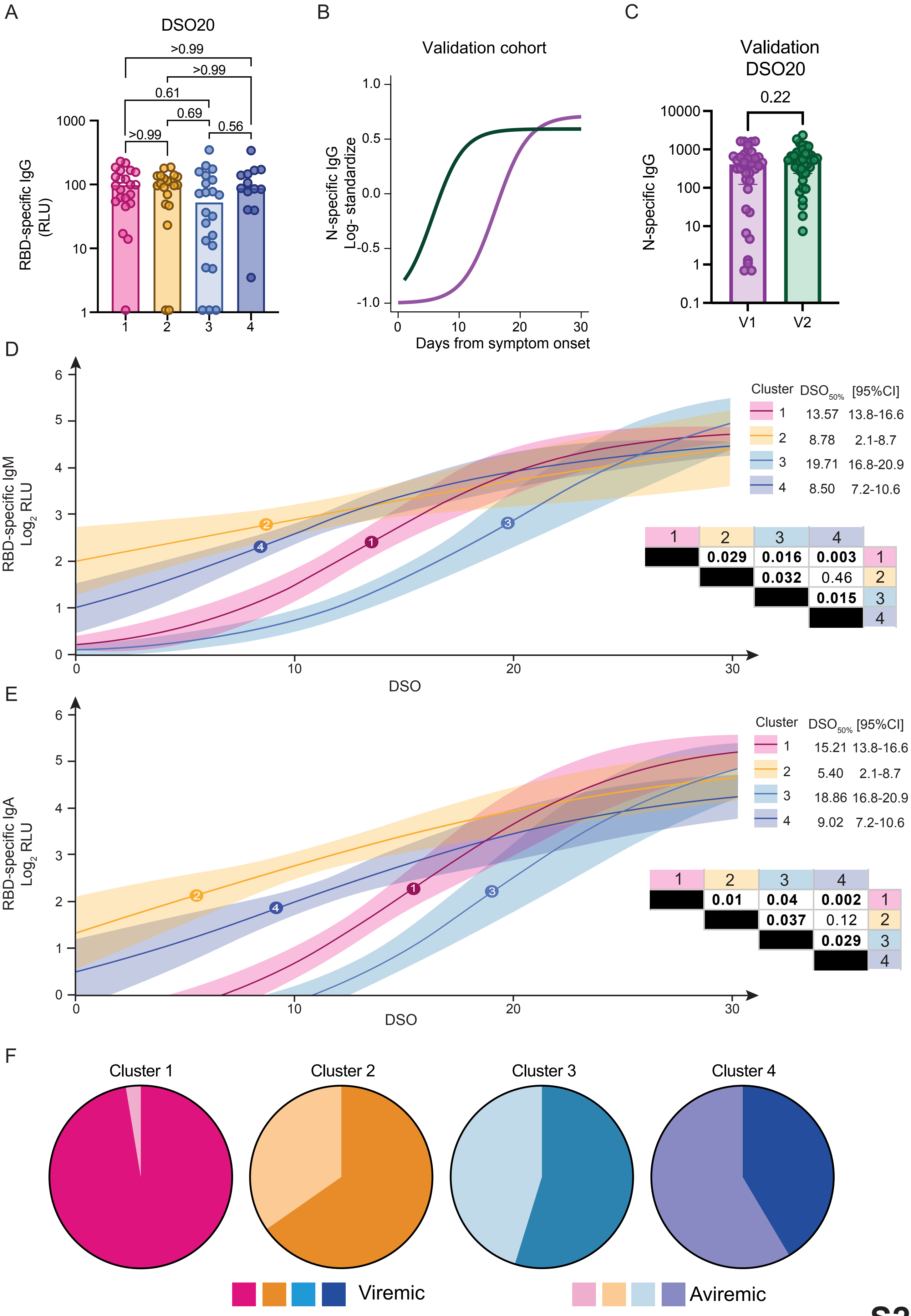
A
DSO20
>0.99
>0.99
0.61
0.69
>0.99
0.56
RBD-specific IgG (RLU)
B
Validation cohort
N-specific IgG Log- standardize
Days from symptom onset
C
Validation DSO20
0.22
N-specific IgG
V1
V2
D
RBD-specific IgM Log2 RLU
DSO
Cluster DSO50% [95%CI]
1 13.57 13.8-16.6
2 8.78 2.1-8.7
3 19.71 16.8-20.9
4 8.50 7.2-10.6
0.029 0.016 0.003
0.032 0.46
0.015
E
RBD-specific IgA Log2 RLU
DSO
Cluster DSO50% [95%CI]
1 15.21 13.8-16.6
2 5.40 2.1-8.7
3 18.86 16.8-20.9
4 9.02 7.2-10.6
0.01 0.04 0.002
0.037 0.12
0.029
F
Cluster 1
Cluster 2
Cluster 3
Cluster 4
Viremic
Aviremic

S2

Supplementary Figure 2. Kinetics of RBD-specific antibody responses per patient clusters. **A)** RBD-specific IgG levels at DSO20 (+/- 4 days) per patient cluster. **B)** Sigmoidal curve fitted to the average per day per patient cluster of RBD-specific IgG responses, along day since symptom onset, within the validation cohort. **C)** RBD-specific IgG levels at DSO20 (+/- 4 days) per patient cluster of the validation cohort. **DE)** Model and statistical comparisons of RBD-specific D) IgM and E) IgA of the discovery cohort. **F**) Fraction of patients per cluster with at least one sample with detectable plasma vRNA (>13 copies/mL, saturated pie slices) throughout hospital stay. N discovery cohort: 1 = 38; 2 = 49; 3 = 73; 4 = 82 (242 in total). N validation cohort: V1 = 37; V2 = 39 (76 in total). A) Kruskal-Wallis with Dunn's multiple comparison tests. B) Mann-Whitney test. DE) Two-stage bootstrap, with 1000 simulations. Pairwise comparisons between all four clusters.

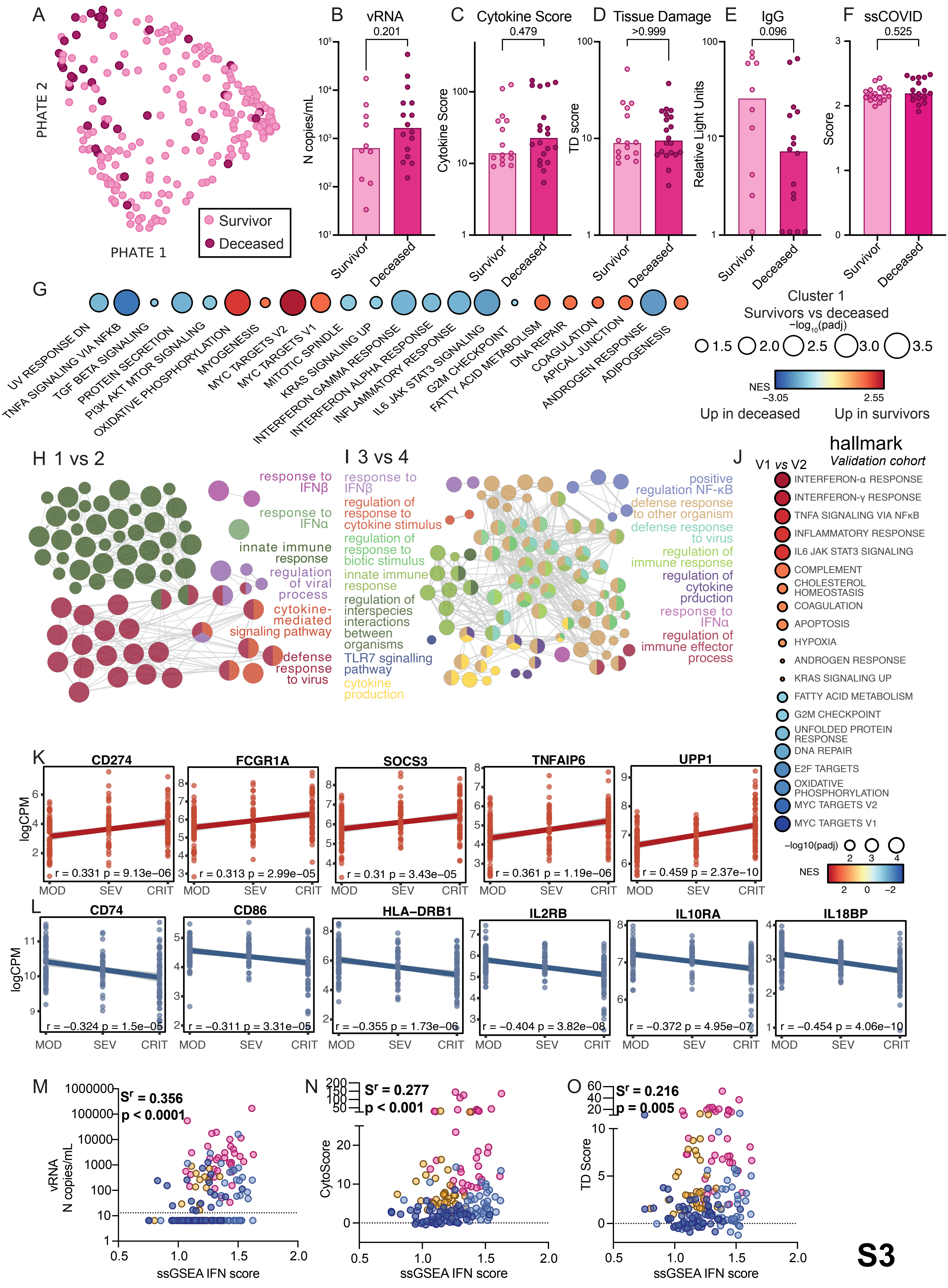

A
PHATE 2
PHATE 1
Survivor
Deceased
B vRNA
0.201
N copies/mL
C Cytokine Score
0.479
D Tissue Damage
>0.999
TD score
E IgG
0.096
Relative Light Units
F ssCOVID
0.525
Score
Survivor
Deceased
G
UV RESPONSE DN
TNFA SIGNALING VIA NFKB
TGF BETA SIGNALING
PROTEIN SECRETION
PI3K AKT MTOR SIGNALING
OXIDATIVE PHOSPHORYLATION
MYOGENESIS
MYC TARGETS V2
MYC TARGETS V1
MITOTIC SPINDLE
KRAS SIGNALING UP
INTERFERON GAMMA RESPONSE
INTERFERON ALPHA RESPONSE
INFLAMMATORY RESPONSE
IL6 JAK STAT3 SIGNALING
G2M CHECKPOINT
FATTY ACID METABOLISM
DNA REPAIR
COAGULATION
APICAL JUNCTION
ANDROGEN RESPONSE
ADIPOGENESIS
Cluster 1
Survivors vs deceased
−log10(padj)
1.5 2.0 2.5 3.0 3.5
NES
−3.05 2.55
Up in deceased
Up in survivors
H 1 vs 2
response to IFNβ
response to IFNα
innate immune response
regulation of viral process
cytokine-mediated signaling pathway
defense response to virus
I 3 vs 4
response to IFNβ
regulation of response to cytokine stimulus
regulation of response to biotic stimulus
innate immune response
regulation of interspecies interactions between organisms
TLR7 singalling pathway
cytokine production
positive regulation NF-κB
defense response to other organism
defense response to virus
regulation of immune response
regulation of cytokine prduction
response to IFNα
regulation of immune effector process
J
hallmark
Validation cohort
V1 vs V2
INTERFERON-α RESPONSE
INTERFERON-γ RESPONSE
TNFA SIGNALING VIA NFκB
INFLAMMATORY RESPONSE
IL6 JAK STAT3 SIGNALING
COMPLEMENT
CHOLESTEROL HOMEOSTASIS
COAGULATION
APOPTOSIS
HYPOXIA
ANDROGEN RESPONSE
KRAS SIGNALING UP
FATTY ACID METABOLISM
G2M CHECKPOINT
UNFOLDED PROTEIN RESPONSE
DNA REPAIR
E2F TARGETS
OXIDATIVE PHOSPHORYLATION
MYC TARGETS V2
MYC TARGETS V1
−log10(padj) 2 3 4
NES 2 0 −2
K
CD274
r = 0.331 p = 9.13e−06
FCGR1A
r = 0.313 p = 2.99e−05
SOCS3
r = 0.31 p = 3.43e−05
TNFAIP6
r = 0.361 p = 1.19e−06
UPP1
r = 0.459 p = 2.37e−10
logCPM
MOD SEV CRIT
L
CD74
r = −0.324 p = 1.5e−05
CD86
r = −0.311 p = 3.31e−05
HLA−DRB1
r = −0.355 p = 1.73e−06
IL2RB
r = −0.404 p = 3.82e−08
IL10RA
r = −0.372 p = 4.95e−07
IL18BP
r = −0.454 p = 4.06e−10
M
Sr = 0.356
p < 0.0001
vRNA N copies/mL
N
Sr = 0.277
p < 0.001
CytoScore
O
Sr = 0.216
p = 0.005
TD Score
ssGSEA IFN score

S3

Supplementary Figure 3. Similar plasma profiles at DSO11 between different outcomes of cluster 1 patients. A) Patients from PHATE embedding color-coded by outcome at DSO60, where mauve dots are fatal outcome, and pink dots are survivors. **B-F)** DSO11 levels, between survivors and non-survivors, of **B**) plasma vRNA; **C**) Cytokine score ; **D**) Tissue damage score and **E**) RBD-specific IgG ; F) ssGSEA COVID severity score. **G**) GSEA on Hallmark gene sets for the contrast survivor vs deceased within cluster 1 only. **H-I)** Gene ontology (GO) enrichments for the Biological Processes (BP) database for significantly upregulated (FDR < 0.01, logFC > 0) genes for contrasts H) 1 vs 2 or I) 3 vs 4. Only significant enrichments (FDR < 0.05) are shown. **J)** Gene set enrichment analysis on differentially-expressed genes between cluster V1 and V2 in validation cohort, using the Hallmark gene sets. Only significant pathways (adjusted p < 0.1) are shown. The size of the circle is representative of the adjusted p value, while the color gives the direction of the enrichement : red means enriched in cluster V1 ; blue is an enrichment in cluster V2. **KL)** Interferon-related genes significantly (|Pearson's r| > 0.3, p < 1 x 10-4) associated with patient severity at the time of sampling. K) genes positively correlated with severity (r > 0); L) genes negatively correlated with severity (r < 0). Pearson r and p values are included in the bottom of the the respective plot. **M-O**) At DSO11, correlation between IFN score and contemporaneous **M**) vRNA; **N**) Cytokine Score or **O**) Tissue damage score. BCDEFMNO) N: 1 = 38; 2 = 49 ; 3 = 73, 4 = 82 (242 in total). GHIKL). N: 1 = 37 ; 2 = 35 ; 3 = 41, 4 = 61 (174 in total). J) N: V1 = 18 ; V2 = 14 (32 in total). BCDEF) Mann-Whitney test. KL) Pearson's correlation. MNO) Spearman correlation.

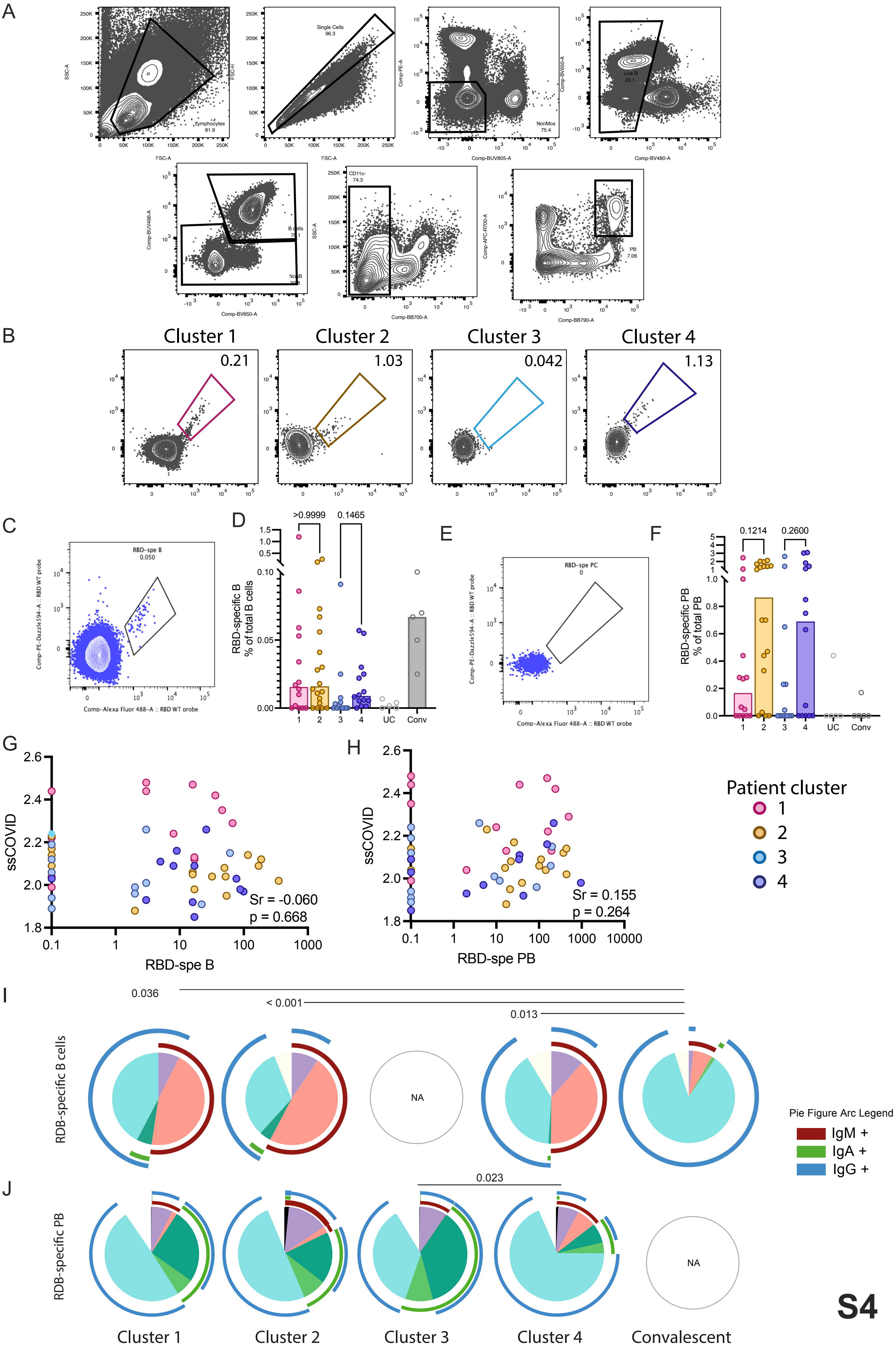

A
Lymphocytes 81.9
Single Cells 96.3
NonMos 75.4
Live B 20.1
B cells 78.1
NonB
CD11c- 74.3
PB 7.06
FSC-A
SSC-A
FSC-H
Comp-PE-A
Comp-BUV805-A
Comp-BV650-A
Comp-BV480-A
Comp-BUV496-A
Comp-BB700-A
Comp-APC-R700-A
Comp-BB790-A
B
Cluster 1
0.21
Cluster 2
1.03
Cluster 3
0.042
Cluster 4
1.13
C
RBD-spe B 0.050
Comp-PE-Dazzle594-A :: RBD WT probe
Comp-Alexa Fluor 488-A :: RBD WT probe
D
>0.9999
0.1465
RBD-specific B % of total B cells
1
2
3
4
UC
Conv
E
RBD-spe PC 0
F
0.1214
0.2600
RBD-specific PB % of total PB
G
ssCOVID
RBD-spe B
Sr = -0.060
p = 0.668
H
RBD-spe PB
Sr = 0.155
p = 0.264
Patient cluster
1
2
3
4
I
0.036
< 0.001
0.013
RDB-specific B cells
NA
Pie Figure Arc Legend
IgM +
IgA +
IgG +
J
0.023
RDB-specific PB
NA
Cluster 1
Cluster 2
Cluster 3
Cluster 4
Convalescent

S4

Supplementary Figure 4. Distribution of isotypes among RBD-specific antibody secreting cells. A) Representative gating strategy to identify B cells (CD19+ CD20+) or plasmablasts (CD19+/- CD20- CD11c- CD38+ CD27+). **B)** Representative gating strategy of RBD-specific PB per cluster. **C)** RBD gate on B cells from a convalescent subject. **D)** Frequency of B cells which are RBD-specific in all four acute COVID-19 clusters, uninfected donors (UC) or convalescent subjects (Conv). **E)** RBD gate on plasma cells from a convalescent subject. **F)** Frequency of PB cells which are RBD-specific in all four acute COVID-19 clusters, uninfected donors (UC), or convalescent subjects (Conv). n for cluster 1 = 14; 2 = 16; 3 = 12; 4 = 13; Conv = 3; UC = 5. **GH)** Correlation between ssGSEA COVID-19 severity score and absolute counts of RBD-specific G) B cells or H) PB. **IJ)** Pie chart of isotype expression among I) RBD-specific B cells or J) RBD-specific PB per patient clusters. n for cluster 1 = 14; 2 = 16; 3 = 12; 4 = 13. DF) Kruskal-Wallis with Dunn's multiple comparison tests. GH) Spearman correlations. IJ) Permutation test (1000 iterations).

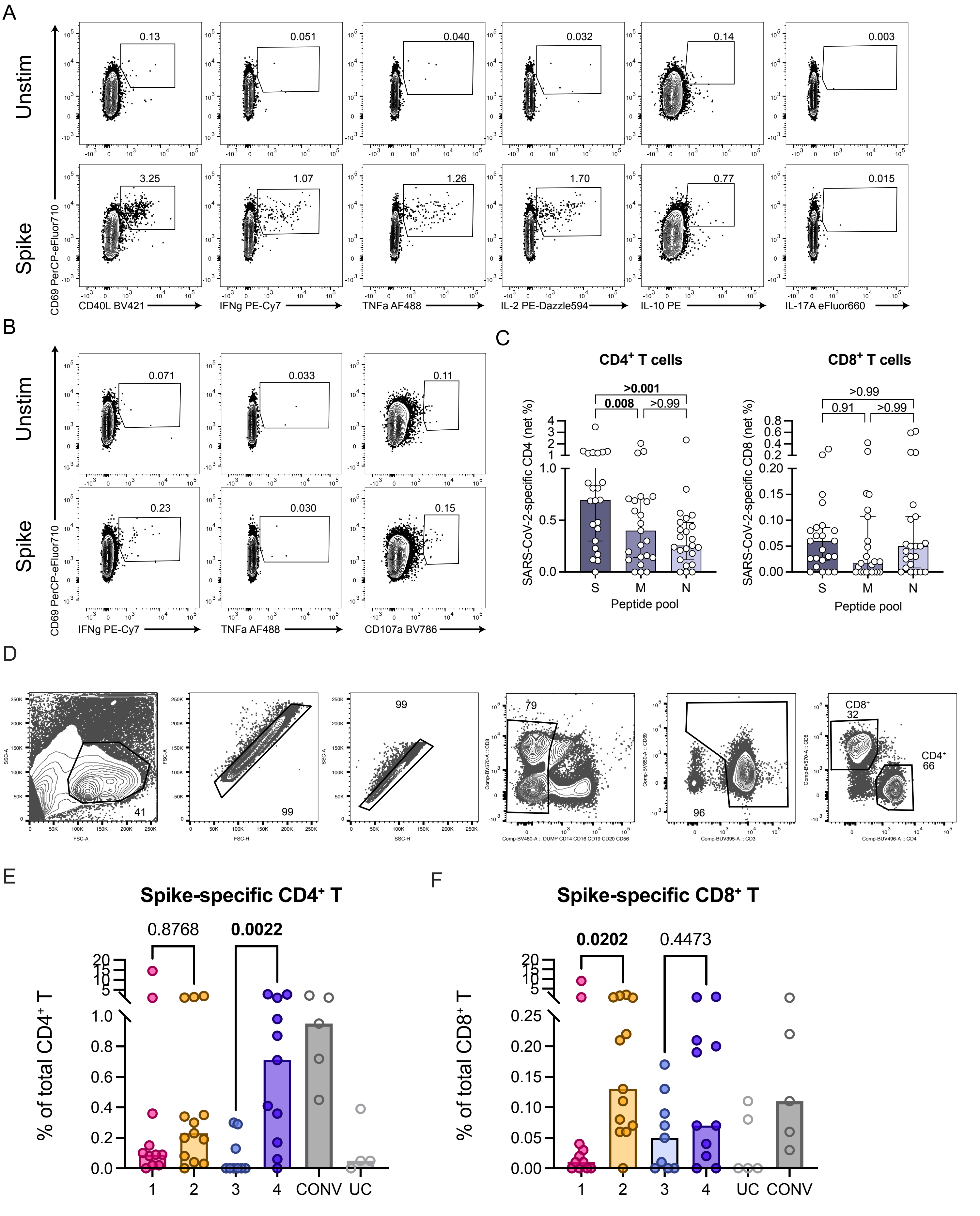

A
Unstim
Spike
CD69 PerCP-eFluor710
0.13
0.051
0.040
0.032
0.14
0.003
3.25
1.07
1.26
1.70
0.77
0.015
CD40L BV421
IFNg PE-Cy7
TNFa AF488
IL-2 PE-Dazzle594
IL-10 PE
IL-17A eFluor660
B
0.071
0.033
0.11
0.23
0.030
0.15
IFNg PE-Cy7
TNFa AF488
CD107a BV786
C
CD4+ T cells
CD8+ T cells
>0.001
0.008
>0.99
>0.99
0.91
>0.99
SARS-CoV-2-specific CD4 (net %)
SARS-CoV-2-specific CD8 (net %)
S
M
N
Peptide pool
D
41
99
99
79
96
CD8+ 32
CD4+ 66
FSC-A
SSC-A
FSC-H
SSC-H
Comp-BV480-A :: DUMP CD14 CD16 CD19 CD20 CD56
Comp-BUV395-A :: CD3
Comp-BUV496-A :: CD4
Comp-BV570-A :: CD8
Comp-BV650-A :: CD69
E
Spike-specific CD4+ T
0.8768
0.0022
% of total CD4+ T
1
2
3
4
CONV
UC
F
Spike-specific CD8+ T
0.0202
0.4473
% of total CD8+ T
1
2
3
4
UC
CONV

S5

Supplementary Figure 5. Spike-specific T cell responses in acute and resolved COVID-19. **A)** PBMCs from acutely-SARS-CoV-2-infected individuals were stimulated with peptide pools of viral antigens (Spike – S , M or N) for 6hrs (addition of BFA 1 hour after stimulation). Representative gates on cytokine+ $CD4^+$ or $CD8^+$ T cells. **B)** Net frequency of SARS-CoV-2-specific $CD4^+$ T cells, as calculated using a Boolean OR gate on CD69 vs CD40L, IFNγ, IL-2, IL-10, IL-17, or TNFα. **C)** Net frequency of SARS-CoV-2-specific $CD8^+$ T cells, as calculated using a Boolean OR gate on CD69 vs CD107a, IFNγ or TNFα. **D**) Following 9hrs stimulation of PBMCs with Spike peptide pool, representative gating strategy for identify $CD4^+$ or $CD8^+$ T cells using AIM panel. **EF)** Frequency of **E**) $CD4^+$ T or **F**) $CD8^+$ T cells which are Spike-specific in all four acute COVID-19 clusters, uninfected donors (UC), or convalescent subjects (Conv). C) n = 26 COVID-19+ acute samples (3 were excluded due to incomplete sampling). EF) n for cluster 1 = 11; 2 = 13; 3 = 9; 4 = 11; Conv = 5; UC = 5. C) Friedman test, with Dunn's multiple comparison test. EF) Kruskal-Wallis with Dunn's multiple comparison tests.

Supplementary Table 1: Plasma analytes measured in the discovery and validation cohorts.

Analyte	Discovery	Validation
TNF		
CXCL13		not included
IL-6		
IL-23		not included
CXCL8/IL-8		
CCL2		
IL-1Ra		
Angiopoietin-2		not included
RAGE		
Surfactant Protein D		not included
IgG		
IgA		
IgM		
vRNA		

Supplementary Table 2. Number of viremic participants, with their respective sampling counts, in the discovery cohort.

	Number of observations	Unique patients
Cluster 1	86	32
Cluster 2	49	20
Cluster 3	55	27
Cluster 4	34	18
Total	224	97

Supplementary Table 3: Flow cytometry panel used to detect RBD-specific B and PC

Antigen/Reagent	Fluorochrome	Clone	Manufacturer	Cat #	Dilution
Brilliant Stain buffer	-	-	BD		1:4
LIVE/DEAD	Efluor-506	-	Invitrogen	65-0866	1:200
CD5	BUV395	UCHT1	BD	563546	1:100
CD20	BUV496	2H7	BD	749954	1:20
IgD	BUV563	IA6-2	BD	741394	1:100
CD138	BUV661	MI15	BD	749873	1:100
IgM	BUV737	UCH-B1	Bd	748928	1:200
CD14	BUV805	M5E2	BD	612902	3:100
IgG	BV421	G18-145	BD	562581	1:50
CD3	BV510	UCHT1	BD	566105	1:200
CD56	BV480	NCAM16.2	BD	566124	1:200
CXCR5**	BV605	J252D4	Biolegend	356929	1:25
CD19	BV650	SJ25C1	Biolegend	363028	1:100
T-BET ***	BV711	O4-46	BD	563320	1:20
CD21	BV786	B-LY4	BD	740969	1:200
RBD probe	AF488		*In house*	NA	1:250
CD11c	BB700	SHCL-3	BD	746106	1:100
CD38	BB790	HIT2	BD	*custom*	3:100
RBD probe	AF594		*In house*	NA	1:250
Ki67***	PE-Cy7	B56	BD	561283	1:20
IgA	APC Vio770	IS11-8E10	Miltenyi	130-113-999	1:40
CD27	APC-R700	M-T271	BD	566116	1:200
S100A8/9***	eF660	CF-145	Ebioscience	50-9745-42	1:20

* one test : 5M PBMC in 100µL staining buffer
** added in cull culture (0.5ml) 15 min prior to cell collection for staining.
***intranuclear or intracellular staining

Supplementary Table 4: Flow cytometry panel used to detect AIM⁺ Spike-specific T cells

Antigen/Reagent	Fluorochrome	Clone	Manufacturer	Cat #	Dilution
Brilliant Stain buffer	-	-	BD	563794	1:4
LIVE/DEAD	Efluor-506	-	Invitrogen	65-0866	1:200
CD40 block**	-	HB14	Miltenyi	130-094-133	1:100
CD3	BUV395	UCHT1	Bd	563546	3:100
CD4	BUV496	SK3	BD	612936	1:25
CD27	BUV661	L128	BD	750167	1:200
CCR6**	BUV737	11A9	BD	564377	1:200
CXCR6**	BUV805	13B 1E5	BD	748448	1:50
CXCR5**	BV421	J252D4	Biolegend	356920	3:100
CD14	BV480	M5E2	BD	746304	3:100
CD19	BV480	HIB19	BD	746457	3:100
CD16	BV480	3G8	BD	566108	1:100
CD20	BV480	2G7	BD	566181	1:200
CD56	BV480	NCAM16.2	BD	566124	1:200
CD8	BV570	RPA-T8	Biolegend	301037	1:100
CXCR3**	BV605	G025H7	Biolegend	353728	1:200
CD69	BV650	FN50	Biolegend	310934	1:50
PD-1	BV711	EH12.2H7	Biolegend	329928	1:25
HLA-DR	FITC	LN3	Biolegend	327005	1:200
CD45RA	PerCP Cy5.5	HI100	BD	563429	1:200
CD38	BB790	HIT2	BD	*custom*	3:100
CD40L	PE	TRAP1	BD	555700	1:20
41BB	PE-Dazzle594	4B4-1	Biolegend	309826	1:50
CCR7**	PE-Cy7	3D12	BD	560922	1:100
OX40	APC	ACT35	BD	563473	1:50

* one test : 1.7M PBMC in 100µL staining buffer or 170µL of culture media
** added in cull culture (0.5mL) 15 min prior to stimulation

Supplementary Table 5. Flow cytometry antibody staining panel for intracellular cytokine detection

Target	Fluorochrome	Clone	Manufacturer	Detection	Catalog number	Dilution
CD3	BUV395	UCHT1	BD	Surface	563546	3:100
CD4	BUV496	SK3	BD	Surface	564651	1:25
CD8	BV570	RPA-T8	Biolegend	Surface	344732	1:100
CD14	BUV805	M5E2	BD	Surface	612902	3:100
CD16	BV650	3G8	Biolegend	Surface	302042	3:100
CD19	APC-eFluor780	HIB19	ThermoFisher	Surface	47-0199-42	1:200
CD56	BUV737	NCAM16.2	BD	Surface	564448	1:40
CD69	PerCP-eFluor710	FN50	ThermoFisher	Intracellular	46-0699-42	1:25
CD107a**	BV785	H4A3	Biolegend	During stimulation	328644	1:100
CD154** (CD40L)	BV421	TRAP1	BD	Intracellular	563886	1:20
Granzyme B**	AF700	GB11	BD	Intracellular	561016	1:100
IFN-γ**	PE-Cy7	B27	BD	Intracellular	557643	1:20
IL-2**	PE-Dazzle594	MQ1-17H12	Biolegend	Intracellular	500344	3:100
IL-10**	PE	JES3-9D7	BD	Intracellular	554498	1:20
IL-17A**	eFluor660	eBio64CAP17	ThermoFisher	Intracellular	50-7178-42	1:20
TNF-α**	AF488	MAb11	Biolegend	Intracellular	502915	1:50

*One test: 2M PBMCs in 100µl staining buffer for surface and intracellular detection or 2M PBMCs in 500µl of media for staining during culture
**intranuclear or intracellular staining
