# Supplementary material for: Sustained IFN signaling is associated with delayed development of SARS-CoV-2-specific immunity
Source: Nat Commun. 2024 May 16;15:4177. doi: 10.1038/s41467-024-48556-y (PMC11522391; doi:10.1038/s41467-024-48556-y)
Supplement: Supplementary file 3 — Description of additional supplementary files [file 41467_2024_48556_MOESM3_ESM.pdf]

DESCRIPTION OF ADDITIONAL SUPPLEMENTARY FILES

File name: Supplementary Data 1

Description: Sample meta data. Description of cohort samples, including demographic information about donors, technical batch/variable information, and experimental information.

File name: Supplementary Data 2

Description: Phate effects. PHATE differential expression effect for each gene in each contrast is reported, including effect size estimates, p-values, FDRs (Benjamini-Hochberg and permutation-based), and t-statistics.

File name Supplementary Data 3

Description: PHATE differential expression enrichments. Gene ontology enrichments for the differential expression effects are reported for the contrasts of interest. Term-specific p-values, FDRs (Benjamini-Hochberg-adjusted), enrichment scores, and leading edge gene sets are included.
