# Supplementary material for: Sustained IFN signaling is associated with delayed development of SARS-CoV-2-specific immunity
Source: Nat Commun. 2024 May 16;15:4177. doi: 10.1038/s41467-024-48556-y (PMC11522391; doi:10.1038/s41467-024-48556-y)
Supplement: Supplementary file 7 — Reporting Summary [file 41467_2024_48556_MOESM7_ESM.pdf]

nature portfolio

Corresponding author(s): Daniel E. Kaufmann, Guy Wolf

Last updated by author(s): Mar 21, 2024

Reporting Summary

Nature Portfolio wishes to improve the reproducibility of the work that we publish. This form provides structure for consistency and transparency in reporting. For further information on Nature Portfolio policies, see our Editorial Policies and the Editorial Policy Checklist.

Statistics

For all statistical analyses, confirm that the following items are present in the figure legend, table legend, main text, or Methods section.

n/a	Confirmed	
☐	☒	The exact sample size (n) for each experimental group/condition, given as a discrete number and unit of measurement
☐	☒	A statement on whether measurements were taken from distinct samples or whether the same sample was measured repeatedly
☐	☒	The statistical test(s) used AND whether they are one- or two-sided<br>*Only common tests should be described solely by name; describe more complex techniques in the Methods section.*
☐	☒	A description of all covariates tested
☐	☒	A description of any assumptions or corrections, such as tests of normality and adjustment for multiple comparisons
☐	☒	A full description of the statistical parameters including central tendency (e.g. means) or other basic estimates (e.g. regression coefficient) AND variation (e.g. standard deviation) or associated estimates of uncertainty (e.g. confidence intervals)
☐	☒	For null hypothesis testing, the test statistic (e.g. F, t, r) with confidence intervals, effect sizes, degrees of freedom and P value noted<br>*Give P values as exact values whenever suitable.*
☒	☐	For Bayesian analysis, information on the choice of priors and Markov chain Monte Carlo settings
☐	☒	For hierarchical and complex designs, identification of the appropriate level for tests and full reporting of outcomes
☐	☒	Estimates of effect sizes (e.g. Cohen's d, Pearson's r), indicating how they were calculated

Our web collection on statistics for biologists contains articles on many of the points above.

Software and code

Policy information about availability of computer code

Data collection	Data collection and compilation was done using excel (vl6.68) and R (see below - bulk RNA Seq section).
Data analysis	For PHATE-related analyses, we used the PHATE Python package (vl.0.9) and the k-means implementation of the scikit-learn Python package (v1.0.2). The MELD package was used (v1.0.0) for some representations in the supplemental figures.<br>Statical testing on analyte measurements and flow cytometry data were performed using Graphpad Prism (v9.5.0) and SPICE (v6) for permutation tests. Statistical models were generated using the following R packages : drc (v3.0-1) and mgcv (v1.9-1).<br>For bulk RNA Seq analysis, we used the following packages : RTA v3.4.4 for base calling ; bcl2fastq2 v2.20 for demultiplexing samples and generating fastq files ; sequencing trimming with CutAdapt ; mapping with 2STAR v2.6.1d ; calculation of normalization factors using calcNormFactors in edgeR (v3.26.8) (edgeR was also used for calculating effect size ; limma (v3.40.6) to apply size factors and regress out technical effect of collection center ; GSVA package (v1.32.0) in R to calculate ssGSEA; fgsea (v1.10.1) and ClueGo (v2.5.7) for GSEA.<br>All scripts and processed data are available on Github : https://github.com/herandolph/COVID-19_PHATE and https://github.com/sachaMorin/covid-plasma-clusters

For manuscripts utilizing custom algorithms or software that are central to the research but not yet described in published literature, software must be made available to editors and reviewers. We strongly encourage code deposition in a community repository (e.g. GitHub). See the Nature Portfolio guidelines for submitting code & software for further information.

Data

Policy information about availability of data

All manuscripts must include a data availability statement. This statement should provide the following information, where applicable:

- Accession codes, unique identifiers, or web links for publicly available datasets
- A description of any restrictions on data availability
- For clinical datasets or third party data, please ensure that the statement adheres to our policy

The plasma analyte measurements (relating to Figures 1 and 2) and the frequency of CoV-2-specific immune cells (relating to Figures 4 and 5) of the discovery cohort, following appropriate batch corrections and normalizations, can be found in Source_Data_File.xlsx. Given that Tables 1 and 2 describe the demographics of our cohorts, we have represented age using age brackets instead of exact values for ethical reasons (to further protect participant identities)

The anonymized minimum dataset of bulk RNA Seq data (without any PHI, or information that could allow identification of individuals in the studies) is available on Zenodo (10.5281/zenodo.6963452). All code used for the analysis of the bulk RNA Seq data is available on Github (https://github.com/herandolph/COVID-19_PHATE). Raw sequencing and transcriptomic data can be accessed after request via the "Biobanque Québécoise de la COVID-19 (BQC-19)" (Quebec COVID-19 Biobank) data repository (info@bqc19.ca ; visit https://en.quebeccovidbiobank.ca for more information about the procedure and the data access agreement).

Data from the Pronmed study (validation cohort) is available from the SciLifeLab data repository after appropriate permissions and data access agreements (https://doi.org/10.17044/scilifelab.14229410).

The human genome hg19 was used for alignement (https://www.ncbi.nlm.nih.gov/datasets/genome/GCF_000001405.13/)

Research involving human participants, their data, or biological material

Policy information about studies with human participants or human data. See also policy information about sex, gender (identity/presentation), and sexual orientation and race, ethnicity and racism.

Reporting on sex and gender	We enrolled participants irrespective of sex or gender. Sex and/or gender were not considered in the study design as selection criteria, as we wanted to have an unbiased view of people hospitalized for COVID, but the information was collected. Sex is here defined as biological sex, based on genotype (23rd pair of chromosomes: XX or XY, based on GWAS analyses. We defined gender as self-reported in the hospital electronic medical record (EMR). In the participants of the present study, there was congruence between both, so we did not perform distinct analyses for sex and gender. We primary refer to sex in our manuscript, given the biological nature of the analyses, the fact that some immune genes are coded by the X chromosome, and well-documented differences in immune responses between men and women.. We compared the proportion of sex between our clusters but saw no differences, so we no longer took into account sex when comparing clusters. However, bulk RNA Seq data was corrected for a number of covariates, including sex. Consent from subject or their legal representant was obtained. In our discovery cohort, 101 patients were female (42%) and 141 patients were male (58%). In our validation cohort, 16 patients were female (21%) and 60 patients were male (79%).
Reporting on race, ethnicity, or other socially relevant groupings	Information regarding self-reported race, ethnicity or other socially relevant groupings were collected as part of the BQC19 protocol (Tremblay et al., Plos One 2021), but were not taken into consideration for this study - as we recruited all patients admitted to the hospital for COVID-19 during our recruitment period. However, we did compare genetic ancestry based on genotypic information (GWAS DNA analyses) between our clusters. As we did not see differences based on ancestry, we did not correct for it.
Population characteristics	A number of population characteristics were collected as part of the BQC19 protocol, but only age, sex, and genotypic data (in the context of defining biological sex and identifying ancestry) were taken into consideration to compare clusters and/or cohorts. Age, sex and BMI were used to correct bulk RNA Seq data.
Recruitment	All patients hospitalized at CHUM or JGH between April 2020 and August 2021 with symptomatic infection, who were enrolled into the BQC19 biobank after informed consent and who met our inclusion criteria, were included into the study. For the validation cohort, the study included patients admitted to intensive care between March 15th, 2020, and July 14th, 2020. All patients had confirmed SARS-CoV-2 by RT-PCR from nasal swab (NSW). As our study only considered patients admitted to hospital, our results may or may not apply to the outpatient population. For participants who presented themselves at the hospital/outpatient clinic for a blood test for the only purpose of biobanking in the BQC19, a compensation for their transportation of $CAD 50.00 was given to them at each follow-up visit, as reimbursement of their transportation costs.
Ethics oversight	The study was approved by the respective IRBs. The COVID participants recruited in Montreal (discovery cohort) were already enrolled in the Quebec COVID-19 Biobank https://www.bqc19.ca/en. We did not separately recruit for the study presented in this manuscript, but used biobanked samples and data from the repository. The multicentric BQC19 study protocol number is MP-02-2020-8929. The local IRB protocol number for our study presented here is 20.169.<br>For the validation cohort, the protocols were approved by the Swedish National Ethical Review Agency (Pronmed study; 2017-043, amended 2019-00169, 2020-01623, 2020-05730 and 2022-00526-01). It is also registered a priori at ClinicalTrials.gov (NCT03720860).

Note that full information on the approval of the study protocol must also be provided in the manuscript.

Field-specific reporting

Please select the one below that is the best fit for your research. If you are not sure, read the appropriate sections before making your selection.

- [x] Life sciences
- [ ] Behavioural & social sciences
- [ ] Ecological, evolutionary & environmental sciences

For a reference copy of the document with all sections, see nature.com/documents/nr-reporting-summary-flat.pdf

Life sciences study design

All studies must disclose on these points even when the disclosure is negative.

Sample size

With regards to the cohort from Montreal, we included all individuals previously prospectively enrolled into the BQC19 biobank and hospitalized for COVID-19 who met our selection criteria, and for which sufficient samples were available to perform all immunomonitoring tests. We selected the number of clusters based on the final number of patients included in the study (see Material and Methods for details). Given the precise timing of sampling duing the course of this very dynamic infection, this sample size was sufficient to identify groups of patients with greater similarity in immune phenotype through unsupervised clustering.

For the validation cohort in Uppsala, we included all patients recruited who met our criteria and for whom immunomonitoring had been performed in the same timeframe as the discovery cohort. While the number of patients was smaller, the spectrum of disease severity was also narrower as only ICU patients were enrolled in the Pronmed study. We were thus able to reduce the number of clusters to two, with groups of patients showing great similarity with the clusters enriched in critical disease in the discovery cohort.

Data exclusions

Among patients that met our criteria, those for whom we did not have a plasma sample available, or for whom only incomplete immunomonitoring could be performed in the narrow timeframe (11 days post symptom onset,+/- 4 days) were excluded. The clustering and visualization methods do not allow for incomplete datasets, and the use of extrapolation methods resulted in artificial clustering of datapoints with incomplete datasets. Indeed, although extrapolation methods were investigated with a subset of the discovery cohort early during data accumulation, they were found to be unsuitable for our study for this reason, and samples with incomplete immunomonitoring data were excluded in the already-acquired and downstream datasets. Finally, as the kinetics of the de novo antibody response to SARS-CoV-2 was a key part of our study design, we excluded patients which had a prior vaccination, or a prior known infection of SARS-CoV-2.

Replication

For the measurement of cytokine and tissue damage markers in plasma, each plasma sample was performed as a duplicate in the multiplexed beads arrays, and the average value was retained. Each plate had a repeated negative control (the same across all plates). A total of 30 samples were repeated in a second experiment and did not show a variation greater than 1 standard deviation.

For SARS-CoV-2 viral RNA measurements, N SARS-CoV-2 quantifications were performed in quadruplicates, and 18S measurements were performed in duplicates. Pre-COVID-19 plasma were included as a negative control. These samples were not repeated.

For the RBD assays, each plasma sample was analyzed in duplicates, and the average value was retained. The antibody CR3022 was included as a positive control, and pre-COVID-19 plasma were included as a negative control. These samples were not repeated.

Whole blood RNA extracts were sequenced once. These samples were not repeated.

Randomization

Samples were accumulated, then a batch of samples would be handed off to the persons doing the experiments to be processed all at once. Sample position on assay plates was randomized (eg. position on an ELISA plate, to avoid batch effects), but this was not practical for low-throughput assays like flow cytometry. The number of samples from controls allocated within batches was not random - we included COVID-19 negative individuals (both hospitalized or healthy controls) in all batches to serve as internal controls.

Blinding

The category of the patient corresponding to the sample (i.e. whether it was from a COVID-19 positive or negative individual, disease severity) was not known to the persons performing the experiments. Results were sent back using anonymized codes. Data compilation was performed separately for plasma measurements and clinical data, thus also maintaining blinding of the operators during this process.

Reporting for specific materials, systems and methods

We require information from authors about some types of materials, experimental systems and methods used in many studies. Here, indicate whether each material, system or method listed is relevant to your study. If you are not sure if a list item applies to your research, read the appropriate section before selecting a response.

Materials & experimental systems

n/a	Involved in the study	
☐	☒	Antibodies
☒	☐	Eukaryotic cell lines
☒	☐	Palaeontology and archaeology
☒	☐	Animals and other organisms
☐	☒	Clinical data
☒	☐	Dual use research of concern
☒	☐	Plants

Methods

n/a	Involved in the study	
☒	☐	ChIP-seq
☐	☒	Flow cytometry
☒	☐	MRI-based neuroimaging

Antibodies

Antibodies used

The monoclonal antibody CR3022 was used as a positive control in the RBD ELISA assay. This antibody was provided by Dr M. Gordon Joyce, and it's identifier is : RRID:AB_2848080.

All antibodies used in the flow cytometry assays were primary antibodies, and listed in Tables S4 to S6.

For the detection of RBD-specific B cells and PC, we purchased the following antibodies from BD Biosciences : CD5 BUV395 (clone UCHT1, cat# : 563546, dilution 1:100) ; CD20 BUV496 (clone 2H7, cat #: 749954, dilution 1:20) ; IgD BUV563 (clone: IA6-2, cat#: 741394, dilution 1:100) ; CD138 BUV661(clone: MI15, cat#: 749873, dilution 1:100) ; IgM BUV737 (clone: UCH-B1, cat#: 748928, dilution 1:200) ; CD14 BUV805 (clone: M5E2, cat#: 612902, dilution : 3:100) ; IgG BV421 (clone: G18-145, cat#: 562581, dilution 1:50) ; CD3 BV510 (clone: UCHT1, cat#: 566105, dilution 1:200) ; CD56 BV480 (clone: NCAM16.2, cat# : 566124, 1:200) ; T-BET BV711 (clone: O4-46, cat#: 563320, 1:20) ; CD21 BV786 (clone: B-LY4, cat#: 740969, 1:200) ; CD11c BB700 (clone: SHCL-3, cat#: 746106, 1:100) ; CD38 BB790 (clone: HIT2, 3:100) ; Ki67 PE-Cy7 (clone: B56, cat#: 561283, 1:20) ; CD27 APC-R700 (clone : M-T271, cat#: 566116, 1:200). Additional antibodies on this panel were purchased from other companies : CXCR5 BV605 (clone: J252D4, manufacturer: Biolegend, cat#: 356929, dilution : 1:25) ; CD19 BV650 (clone: SJ25C1, manufacturer: Biolegend, cat#: 363028, 1:100) ; IgA APC Vio770 (clone: IS11-8E10, manufacturer: Miltenyi, cat# : 130-113-999, 1:40) ; S100A8/9 eF660 (clone: CF-145, manufacturer: Ebioscience, cat#: 50-9745-42, 1:20). Both probes to detect RBD-specificty were generated and validated in-house (by the team of Dr. Andres Finzi) : RBD probe AF488, dilution 1:250 ; RBD probe AF594, 1:250.

For the detection of AIM+ spike-specific T cells, we purchased the following primary antibodies from BD Biosciences: CD3 BUV395 (clone: UCHT1, cat#: 563546, 3:100) ; CD4 BUV496 (clone: SK3, cat#: 612936, 1:25); CD27 BUV661 (clone: L128, cat#: 750167, 1:200); CCR6 BUV737 (clone: 11A9, cat#: 564377, 1:200); CXCR6 BUV805 (clone: 13B 1E5, cat#: 748448, 1:50); CD14 BV480 (clone: M5E2, cat#: 746304, 3 :100) ; CD19 BV480 (clone: HIB19, cat#: 746457, 3:100) ; CD16 BV480 (clone: 3G8, cat#: 566108, 1:100) ; CD20 BV480 (clone: 2G7, cat#: 566181, 1:200) ; CD56 BV480 (clone: NCAM16.2, cat#: 566124, 1:200) ; CD45RA PerCP Cy5.5 (clone: HI100, cat#: 563429, 1:200); CD38 BB790 (clone: HIT2, cat#: 566445); CD40L PE (clone: TRAP1, cat#: 555700, 1:20) ; CCR7 PE-Cy7 (clone: 3D12, cat#: 560922, 1:100) ; OX40 APC (clone: ACT35, cat#: 563473, 1:50). The following primary antibodiesd were purchased from Biolegend : CXCR5 BV421 (clone : J252D4, cat#: 356920, 3:100); CD8 BV570 (clone: RPA-T8, cat#:301037, 1:100) ; CXCR3 BV605 (clone: G025H7, cat#: 353728, 1:200); CD69 BV650 (clone: FN50, cat#: 310934, 1:50); PD-1 BV711 (clone: EH12.2H7. cat#: 329928, 1:25) ; HLA-DR FITC (clone: LN3, cat#: 327005, 1:200); 41BB PE-Dazzle594 (clone: 4B4-1, cat#: 309826, 1:50). The antibody used to block downregulation of CD40L was CD40 block (clone HB14, manufacturer : Miltenyi, cat# : 130-094-133, 1:100).

For the detection of intracellular staining in T cells upon antigen stimulation, we used the following primary antibodies from BD Biosciences : CD3 BUV395 (clone: UCHT1, cat#: 563546, 3:100) ; CD4 BUV496 (clone: SK3, cat#: 564651, 1:25); CD154 BV421 (clone: TRAP1, cat#: 563886, 1:20) ; CD14 BUV805 (clone: M5E2, cat#: 612902, 3:100) ; CD56 BUV737 (clone: NCAM16.2, cat#: 564448, 1:40) ; Granzyme B AF700 (clone: GB11, cat#: 561016, 1:100) ; IFN-γ PE-Cy7 (clone: B27, cat#: 557643, 1:20) ; IL-10 PE (clone; JES3-9D7, cat#: 554498, 1:20). From Biolegend, we used : CD107a BV785 (clone: H4A3, cat#: 328644, 1 : 100) ; CD8 BV570 (clone: RPA-T8, cat#: 344732, 1:100) ; TNF-α AF488 (clone: MAb11, cat#: 502915, 1:50) ; IL-2 PE-Dazzle594 (clone: MQ1-17H12, cat# : 500344. 3:100) ; CD16 BV650 (clone : 3G8, cat#: 302042, 3:100). We purchased from ThermoFisher : CD19 APC-eFluor780 (clone: HIB19, cat#: 47-0199-42, 1:200) ; IL-17A eFluor660, clone: eBio64CAP17, cat#: 50-7178-42, 1:20), and CD69 PerCPeFluor710 (clone: FN50, cat#: 46-0699-42, 1:40).

Validation

CR3022's ability to bind RBD was show in Yuan et al., Science 2020 (ref 47).

The RBD probes are purified SARS-CoV-2 Spike RBD recombinant protein expressed in Freestyle 293F cells. Their specificity was tested on uninfected controls (PBMCs collected prior to pandemic) as negative controls, and on recovered convalescent patients (infection confirmed by NSW PCR, and sampled after they had cleared the virus) as positive controls (see supplemental figure 4).

All purchased antibodies were validated by the manufacturer and titrated with biological and/or isotype controls.

BD Biosciences has the following statement : "The specificity is confirmed using multiple methodologies that may include a combination of flow cytometry, immunofluorescence, immunohistochemistry or western blot to test staining on a combination of primary cells, cell lines or transfectant models". For more information, please visit : https://www.bdbiosciences.com/en-us/products/reagents/flow-cytometry-reagents/research-reagents/quality-and-reproducibility

Biolegend has the following statement : "Antibody validation is a critical step in the journey towards obtaining consistent reproducibility in science. To ensure they are both specific and sensitive, we validate our antibodies through a variety of methods including: 1) Testing on multiple cell and tissue types with a variety of known expression levels ; 2) Validation in multiple applications as a cross-check for specificity and to provide additional clarity for researchers ; 3) Comparison to existing antibody clones ; 4) Using cell treatments to modulate target expression, such as phosphatase treatment to ensure phospho-antibody specificity. For more information, please visit : https://www.biolegend.com/nl-nl/bio-bits/highly-specific-validated-antibodies#:~:text=Antibody%20validation%20is%20a%20critical,variety%20of%20known%20expression%20levels.

ThermoFisher employ these two methods to assure specificity : 1)" Expanded specificity testing methods to ensure superior antibody results" ; 2) "Improved reporting of antibody specifics such as clone, isotype identity, and functional characteristics to facilitate the replication of published results". For more information, please visit : https://www.thermofisher.com/us/en/home/life-science/antibodies/invitrogen-antibody-validation.html

Miltenyi has the following statement : "All our antibodies are rigorously tested and validated before release. In the application section on the product page, you can find examples of typical performance data. In addition, we provide extended validation data highlighting details of antibody performance, specificity, and fixation compatibility. All antibodies for which any of these datasets are already available will be indicated with the extended validation stamp." For more information, please visit : https://www.miltenyibiotec.com/US-en/products/macs-antibodies/antibody-validation.html

Clinical data

Policy information about clinical studies

All manuscripts should comply with the ICMJE guidelines for publication of clinical research and a completed CONSORT checklist must be included with all submissions.

Clinical trial registration	The BQC19 initiative was not enrolled as a clinical trial, this is a purely observational study (see Tremblay et al., Plos One 2021- ref. 58). The study from which we obtained data for the validation cohort was registered a priori at ClinicalTrials.gov (NCT03720860).
Study protocol	All details for the protocol relating to BQC19 can be found publicly available on the website of the Quebec COVID-19 Biobank: https://www.bqc19.ca/en/availab1e-documents and in Tremblay et al., Plos One 2021 - ref. 58. Details on the trial protocol for the validation cohort can be found at ClinicalTrials.gov (NCT03720860).
Data collection	Individuals comprising the discovery cohort were recruited between April 2020 and August 2021. Most clinical data was obtained through BQC19. Selection of additional relevant data was decided upon a priori early after sample collection, and compiled in parallel from patient charts in an ongoing manner, on-site.<br>Individuals in the validation cohort were recruited between March 2020 and July 2020. Clinical data was compiled at the end of July 2020 on-site.<br>Clinical data were collected within hospital units by the clinical teams and clinical research teams as part of standard patient care (and therefore included in the electronic medical record) or to fill in some specific fields of the case report form.
Outcomes	The primary outcome was all-cause mortality at 60 days post symptom onset. Secondary outcomes were peak disease severity (as measured by level of required respiratory support), and length of stay in hospital.

Plants

Seed stocks	NA
Novel plant genotypes	NA
Authentication	NA

Flow Cytometry

Plots

Confirm that:

- [x] The axis labels state the marker and fluorochrome used (e.g. CD4-FITC).
- [x] The axis scales are clearly visible. Include numbers along axes only for bottom left plot of group (a 'group' is an analysis of identical markers).
- [x] All plots are contour plots with outliers or pseudocolor plots.
- [x] A numerical value for number of cells or percentage (with statistics) is provided.

Methodology

Sample preparation	Detection of RBD-specific B cells<br>Cryopreserved peripheral blood mononuclear cells (PBMCs) were thawed and rested in cell culture media (RPMI supplemented with 10% fetal bovine serum (FBS) and PenStrep - 50 U/ml of penicillin and 50 µg/mL of streptomycin) at 37°C for 3hrs at a density of lx107 cells/ml in 24-well plates. Cells were collected, washed, and stained with LIVE/DEAD'" Fixable Aqua Dead Cell Stain Kit (20 mins, 4°C; Thermofisher, #L34965). After washing, cells were stained with a cocktail of surface markers (30 mins, 4°C; See panel in Supplementary Table 1, including RBD probes). Washed cells were then fixed with 2% paraformaldehyde (PFA) for 20 mins at RT, then washed and resuspended in PBS-2% FBS for flow acquisition on a 5-laser Symphony (BD). Analyses were performed using FlowJo (Treestar, VlO).<br><br>Activation-induced marker (AIM) assay on T cells<br>Cryopreserved PBMCs were thawed and rested in cell culture media (RPMI supplemented with 10% Human AB serum and PenStrep - 50 U/ml of penicillin and 50 µg/ml of streptomycin) at 37°C for 3hrs at a density of lOM/mL in 24-well plates. 15 minutes prior to stimulation, CD40 blocking antibody (clone HB14, Miltenyi, cat#: 130-094-133) was added to each well at 0.5 µg/ml, as well as antibodies staining CXCRS, CXCR3 and CCR6. Cells were either left unstimulated or stimulated with overlapping peptide pools of Spike (Sl + S2), at a final concentration of 0.5 µg/mL/peptide. Alternatively, lµg/ml of Staphylococcal Enterotoxin B (SEB, Toxin Technology) was used to stimulate the cells as a positive control. Cells were

stimulated for 15hrs, collected, washed, and stained with LIVE/DEAD'" Fixable Aqua Dead Cell Stain Kit (20 mins, 4°C; Thermofisher, #L34965). After washing, cells were incubated with FcR block (10mins, 4°C; Miltenyi) then stained with a cocktail of surface markers (30 mins, 4°C; See panel in Supplementary Table 1). Washed cells were then fixed with 2% paraformaldehyde (PFA) for 20 mins at RT, then washed and resuspended in PBS-2% FBS for flow acquisition on a 5-laser Symphony (BD).

Intracellular cytokine staining (ICS) in Spike-specific T cells
Cryopreserved peripheral blood mononuclear cells (PBMCs) were thawed and rested for 2hrs in cell culture media. Cells were stimulated with overlapping peptide pools for SARS-CoV-2 spike (S), membrane (M) and nucleocapsid (NC) (0.5 μ g/ml per peptide from JPT, Berlin, Germany) for 6hrs in the presence of anti-CD107a BV786 (BD Biosciences), Brefeldin A (BD Biosciences) and monensin-1 (BD Biosciences) at 37°C and 5% CO2. DMSO-treated cells served as negative control and SEB-treated cells as positive control. Cells were stained with LIVE/DEAD'" Fixable Aqua Dead Cell Stain Kit (20 mins, 4°C; Thermofisher, #L34965) and surface markers (30 mins, 4°C), followed by detection of intracellular markers using the IC Fixation/Permeabilization kit (Thermo Fisher) according to the manufacturer's protocol before acquisition at 5-laser Symphony (BD) (see Supplementary Table 6 for panel).

Instrument

BD FACSymphony AS

Software

Flow cytometry data was analyzed using FlowJo v10.8.1

Cell population abundance

We did not perform any cell sorts for this study.

Gating strategy

All gating strategies, including FSC/SSC gates of the starting cell population, are shown in Supplementary Figures 4A and SD. Lineage gates were set using FMO controls. Gates on RBD-specific B cells (for S4B) were set on negative controls (PBMC from pre-COVID-19 samples). Gates on AIM+ or cytokine+ T cells were set on the unstimulated condition for the same patient.

☒ Tick this box to confirm that a figure exemplifying the gating strategy is provided in the Supplementary Information.
